# Drug Delivery Based on Stimuli-Responsive Injectable Hydrogels for Breast Cancer Therapy: A Review

**DOI:** 10.3390/gels8010045

**Published:** 2022-01-07

**Authors:** Hai Xin, Sina Naficy

**Affiliations:** 1 Independent Researcher, Hornsby, NSW 2077, Australia; 2 School of Chemical and Biomolecular Engineering, The University of Sydney, Sydney, NSW 2006, Australia; sina.naficy@sydney.edu.au

**Keywords:** injectable hydrogel, breast cancer, stimuli responsiveness, tough hydrogel

## Abstract

Breast cancer is the most common and biggest health threat for women. There is an urgent need to develop novel breast cancer therapies to overcome the shortcomings of conventional surgery and chemotherapy, which include poor drug efficiency, damage to normal tissues, and increased side effects. Drug delivery systems based on injectable hydrogels have recently gained remarkable attention, as they offer encouraging solutions for localized, targeted, and controlled drug release to the tumor site. Such systems have great potential for improving drug efficiency and reducing the side effects caused by long-term exposure to chemotherapy. The present review aims to provide a critical analysis of the latest developments in the application of drug delivery systems using stimuli-responsive injectable hydrogels for breast cancer treatment. The focus is on discussing how such hydrogel systems enhance treatment efficacy and incorporate multiple breast cancer therapies into one system, in response to multiple stimuli, including temperature, pH, photo-, magnetic field, and glutathione. The present work also features a brief outline of the recent progress in the use of tough hydrogels. As the breast undergoes significant physical stress and movement during sporting and daily activities, it is important for drug delivery hydrogels to have sufficient mechanical toughness to maintain structural integrity for a desired period of time.

## 1. Introduction

Breast cancer is the biggest health threat for women, with almost 250,000 identified cases in the United States in 2017. Approximately 12% of women in the U.S. will be diagnosed with breast cancer during their lifetimes [1]. Based on the three biomarkers, estrogen receptor (ER), progesterone receptor (PR), and human epidermal growth factor 2 (HER2), breast cancers are divided into the following three major subcategories: hormone receptor+/HER2−, HER2+, and triple-negative (when none of the three biomarkers is detected). Chemotherapy is a major adjuvant therapy in the HER2+ subtype and is used in combination with endocrine treatment for hormone receptor+ patients. It is applied for women with triple-negative breast cancer [1,2], which is the most challenging subcategory of breast cancer, because of its relatively higher recurrence possibility and lower survival rate [1,2,3]. However, long-term exposure to conventional chemotherapy presents many side effects for breast cancer patients, including vomiting, nausea, fatigue, myelosuppression, nephropathy, peripheral neuropathy, and damage to various important organs due to poor drug efficiency caused by systemic administration [2,4,5,6,7]. Therefore, it is crucial to develop a novel drug delivery system to accomplish more efficient tumor cell eradication and to reduce side effects. To address this challenge, the application of in situ-forming hydrogels that are injected into tumor sites has been gaining increasing interest and attention [8,9,10,11].

Hydrogels are three-dimensional polymeric networks with superior water-absorbing capabilities. Both synthetic polymers and natural polymers have been used for hydrogel preparation, with either chemical or physical crosslinking to form the gel network. Due to their large water content, hydrogels are thought to be biocompatible [12]. With regard to breast cancer, hydrogels composed of nanofibers of cellulose have been demonstrated to embrace the anticancer agent 5-FU to induce the pyroptosis of breast cancer cells [13], and hyaluronic acid hydrogels have been developed to investigate the dormancy of breast cancer cells in brain metastasis [14]. More importantly, hydrogels that release the inhibitor lysine-specific demethylase 1 have been shown to relieve chemoresistance in triple-negative breast cancer treatment [15].

Injectable hydrogels possess all the major characteristics of general hydrogels, and also provide the unique feature of injectability, making them promising candidates for many biomedical applications, such as drug delivery and tissue engineering [16].

Injectability can be achieved by in situ sol–gel transition, where the aqueous solutions of the polymer remain in liquid state but transition to a 3D network at physiological temperatures [17,18]. Another form of injectability is achieved via the shear-thinning behavior of some polymer solutions, in which the gel’s viscosity decreases significantly upon rapid injection and the gel stays at the tumor site following injection [19]. Both types of injectable hydrogel systems will be discussed in the following sections of this review.

Injectable hydrogels are prepared by mixing anticancer drugs with temperature-responsive polymers to form a flowable solution or suspension. Then, the mixture is injected into the tumor site. A sol–gel transition occurs at body temperature, enabling the injected liquid to gelate. The obtained hydrogels remain at the disease site to achieve a sustainable and targeted drug delivery [20,21]. Recently, many works have attempted to incorporate a diverse range of nanoparticles and other functional molecules into the hydrogel depots to fabricate more sophisticated drug delivery platforms that implement combined cancer therapies [22,23,24]. Thus, the modern focus of the injectable hydrogel-based drug delivery system for breast cancer treatment is on creating an integrated system. The system takes advantage of stimuli-responsive polymers and nanoparticles to achieve combined cancer therapies that enhance drug efficacy and decrease side effects.

Although the application of injectable hydrogel-based drug deliveries is well-documented, there are few review articles specifically dedicated to their role in breast cancer treatment. The present work attempts to analyze the most recent research in this special area. The effects of various stimuli, including temperature, pH, photo-, magnetic field, and glutathione on the volume, phase, and structural changes of hydrogel drug delivery will be reviewed. The way in which various therapy approaches, such as chemotherapy, photothermal therapy, photodynamic therapy, and immunotherapy, are combined into one hydrogel network is also discussed. The present work also provides a brief outline of tough hydrogels that may offer encouraging solutions for the mechanical weakness of many conventional hydrogels that have been proposed for drug release. The mechanical integrity of therapeutic hydrogels is an important criterion for applications where the body part, e.g., the breast, undergoes significant stress and displacement during sporting and other physical activities. Therefore, further studies and applications of the special network structures of tough hydrogels are important for researchers who are developing injectable implants to the breast for cancer treatment.

## 2. Stimuli-Responsive Injectable Hydrogels

Table 1 provides a brief summary on some major injectable hydrogel systems that have been prepared for breast cancer therapies. The table also includes four hydrogels prepared to target the cell lines of other cancers. These four hydrogels are cited in the table since they also use Doxorubicin and are stimuli-responsive. They may be inspiring for breast cancer research. The following sections give a more detailed discussion on each of them to explain their applications and treatment effectiveness.

### 2.1. Temperature-Induced Sol–Gel and Gel–Sol Phase Transition

The thermoresponsive polymers with LCST (lower critical solution temperature) around physiological temperature are of great interest, as they remain solution below and gelate above LCST [44]. Regarding the gelation mechanism, above LCST, the miscibility between the macromolecules and water molecules is no longer thermodynamically favorable and polymer–polymer interactions are increased to form physical crosslinks for gel network formation, exhibiting sol–gel transition [16,44,45]. Poly(*N*-isopropylacrylamide) is a well-studied thermoresponsive polymer, and the polymer exhibits a conformational conversion from flexible coil state to globule state near or above LCST [46]. Another big category of thermoresponsive polymers is ABA or BAB triblock copolymers. Segment A is hydrophilic polyethylene and segment B can be polypropylene or polyester including polycarbonate, poly(ε-caprolactone), poly(lactic acid), and poly(lactide-*co*-glycolide) [8]. Above the LCST, the macromolecular chains of these amphiphilic polymers convert to micelles with hydrophilic segment shell and hydrophobic cores [20]. The formation of the micelles may be accompanied by liquid–liquid phase separation where the micelle phase and homogenous phase co-exist [47,48]. In addition to the polymers as mentioned, chitosan derivatives [8,49,50], poly(*N*-vinyl caprolactam) [20,51], and gellan [26] are also reported as thermoresponsive polymers for the injectable hydrogel preparation.

For example, in order to address the breast tumor that may recur and spread distantly, crosslinked chitosan-based hydrogels were studied for delivery of ^131^I-norcholesterol to the mouses models bearing 4T1 breast tumor cell line [52]. Another research group [21] synthesized thermo-responsive injectable poly(lactic acid-co-glycolic acid)-*b*-poly(ethylene glycol)-*b*-poly(lactic acid-*co*-glycolic acid) (PLGA-PEG-PLGA) triblock copolymer hydrogels to deliver the Herceptin antibody to suppress the HER2+ breast tumor and prevent it from worsening.

Instead of simply mixing the anticancer agents with hydrogel polymeric network, many research groups [22,25] have focused on the incorporation of anticancer agents and different nanoparticles into the hydrogel to form a sophisticated hybrid structure to achieve combined therapy induced by temperature responsiveness of the gelling polymers.

For inorganic nanoparticles, the anticancer drug doxorubicin (DOX) was loaded on folic acid-conjugated graphene oxide (GO) particles. The DOX-loaded particles were encapsulated into thermo-responsive hydrogels prepared from grafting copolymerization of chitosan and hyaluronic acid onto poly(*N*-isopropylacrylamide). The product hydrogel had a sol–gel transition temperature around 31 °C and exhibited lessened burst release of DOX from the GO particles. In vivo studies on the MCF-7 breast cancer cells indicated a clear inhibition effect on tumor growth compared to the control groups [25].

In another work [27], tamoxifen citrate (TMC), an anti-breast cancer drug, was made into niosomes with lipid film hydration techniques, and the products were then involved into Pluronic thermoresponsive hydrogels. The gelation temperature of the hydrogel was tuned by varying the ratio of component poloxamers to range between 34 and 37 °C. In vivo animal tests suggested the reduced tumor size was due to the applications of this hydrogel systems to release TMC, compared with free TMC and TMC niosome-alone samples.

In addition to sol–gel transitioning around physiological temperature that occurs immediately after the injection, the thermo-reversible gels that exhibit gel–sol transition induced by the heat after the hydrogel is formed have attracted high attention [53]. For drug delivery, poly(D,L-lactic acid)-poly(ethylene glycol)-poly(D,L-lactic acid) copolymers have been synthesized to incorporate indocyanine green (IG) nanoparticles. Upon NIR light, the hyperthermia generated by IG to 45 °C transferred the gelated PDLLA-PEG-PDLLA back to the sol to facilitate controlled release of resiquimod 484 and CPG ODNs. Both agents initiate immunotherapy to prevent breast cancer recurrence [18]. In another similar work, poly(N-acryloylglycinamide-*co*-acylamide) hydrogel also demonstrated sol–gel–sol reversibility. Upon NIR-induced temperature increase, the gel was converted to sol and it flowed into the breast cavity caused by cancer operation and released the doxorubicin. This process was proposed for the inhibition of breast cancer recurrence [28].

**Figure 1:**
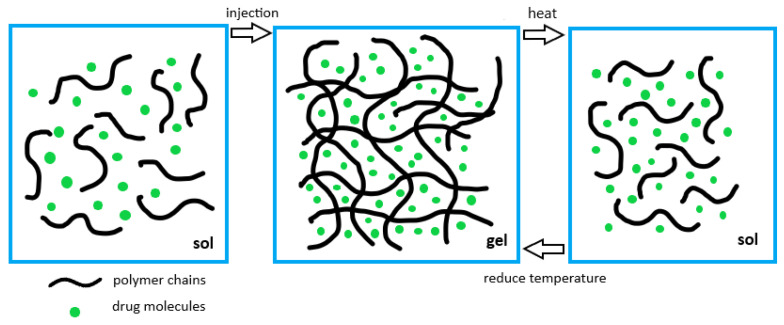
Sol–gel–sol transitions of the injectable hydrogels. The sol turns to gel upon body injection while the formed gel can be converted to sol by local heat.

Figure 1, as shown below, demonstrates the process of sol–gel–sol transition of a hydrogel network that incorporates drug molecules.

In addition to the hydrogels undergoing sol to gel transition around physiological temperature, there is another type of injectability which is realized by the change of hydrogel viscosity in response to the increase in shearing rate and shearing strain. These hydrogels demonstrate shear thinning behaviors and can be injected into the human body and stay at the tumor site after the injection for controlled drug release [19,23,26,40]. In the meantime, some hydrogels of this type also demonstrate self-recovery. Both storage modulus (G′) and loss modulus (G″) show reproducibility in accordance with cyclic increase and decrease in shearing strains [19,23,26].

### 2.2. pH-Dependent Anticancer Drug Release

pH-responsive polymers possess basic or acidic side groups that dissociate or protonate in response to the change of pH [54]. Anionic polymers usually possess –COOH or –SO_3_H groups, and they swell more in basic environment but swell less in an acidic medium [55,56].

**Figure 2:**
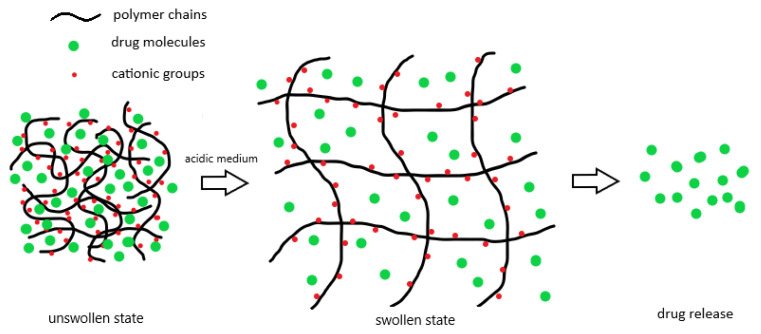
The hydrogels composed of cationic polymers are swollen more in acidic medium to facilitate drug release from their networks.

However, due to the abnormal glucose metabolism, the surrounding environment of the tumor is acidic [57,58]. Therefore, cationic polymers containing basic groups such as –NH_2_ prove more useful for the drug delivery in cancer treatment [54,56]. Once the pH is lower than the dissociation constant, pKa, these pendent groups will protonate as suggested by Henderson–Hasselbalch equation [59,60,61]. As a result, the positively charged polymer chains exhibit electrostatic repulsion against each other to stretch the polymeric network and allow more free space for water migration [61,62]. The basic strength of those side groups and polymeric molecules is definitely a crucial factor to consider the swelling behavior of the hydrogels in the acidic environment [59,60]. Owing to the presence of the charged ions in the network, osmotic pressure also facilitates the water to move into the hydrogel for swelling [61]. The swelling process finally equilibrates with the re-coiling force of the polymer chains, which is closely associated with the polymer elasticity and crosslinking densities [55]. Figure 2 briefly demonstrates the swelling process of a cationic polymer hydrogels in acidic environment.

With regard to injectable hydrogels used for breast cancer treatment, the pH values play crucial roles in determining the drug release profiles [23,43]. More details and discussion are given below.

In one research work, a hydrogel system is developed by mixing oxidized pullulan and chitosan-*g*-dihydrocaffeic acid. In vitro drug release was investigated at 37 °C at the following three different pH levels: 5.5, 6.8 and 7.4. The results illustrate a higher swelling ratio and DOX release percentage at 5.5 than at 7.4, and this was attributed to the electrostatic repulsion of protonated –NH_2_ group in chitosan and weakened bond between –NH_2_ and –CHO formed via Schiff base reaction [29].

Similarly, in another research work, the amino group of glycol chitosan (GC) bonded with the carboxyl group of carboxylated Pluronic F127 to form GC/PF127 micelles, and the micelles were loaded with doxorubicin (DOX). The obtained micelles was used to prepare injectable hydrogels in the presence of α-cyclodextrin via inclusion complexion [31]. In vitro release measurement demonstrated that more DOX was released from the micelles at lower pH. This was ascribed to the protonation of DOX in acidic medium, which promoted its solubility. The positive charges of GC/PF127 hydrogel and subsequent hydrogel dissociation in the acid also contributed to the enhanced release profile [31].

In order to target MCF-7 and MDA-MB-231 cell lines for human breast cancers, a hybrid hydrogel system has been synthesized comprising gelatin and PEG, which were functionalized with hydrazide and aldehyde, respectively. Laponite nanodisks were used as the DOX carrier and embraced within the hydrogel network. The results from in vitro drug release study demonstrated enhanced cumulative release percentage of DOX with decreasing the pH from 7.4 to 5.5 [23]. Again, this observation was attributed to both hybrid hydrogel degradation and increased repulsion between laponite and DOX in acidic environment. Laponite had negatively charged surface and became a competitor against DOX to accept excessive H^+^ in the acidic medium. Such a variation of laponite-DOX electrostatic association in line with the pH change was considered the driving force for extended DOX release as reported [23].

Silk nanofiber was also adopted to prepare injectable hydrogel to suppress MDA-MB-231 breast cancer. As reported, the silk nanofibers with β-sheet conformation formed a physically crosslinked network via self-assembly in aqueous solution and exhibited thixotropic behavior. The antitumor efficacy investigation indicates a reduced tumor weight by the application of this hydrogel system, while drug release tests suggest a higher release rate with the decrease in pH from 7.4 to 6.0 to 4.5 [32].

### 2.3. Photo-Initiated Combinational Breast Cancer Therapy

**Figure 3:**
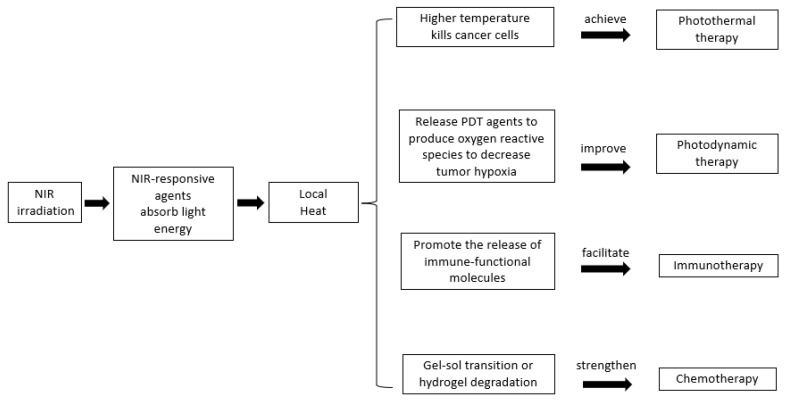
Combinational breast cancer therapies are enabled by embracing NIR-responsive materials into the injectable hydrogel system to enhance treatment efficacy and increase cancer-killing efficiency.

An increasing number of research works show great interest in the application of combinational therapies to realize an enhanced therapeutic efficacy. Two or more therapeutic strategies are integrated into one smart hydrogel system to exert cancer-killing effects [9,11,35]. Some exciting achievements include combined photothermal and photodynamic therapy [11,24], combined photothermal and chemotherapy [63], or combined photothermal and immunotherapy [18]. These combinational cancer treatment approaches contain photo-responsive agents which absorb near infrared (NIR) light to generate heat. The heat generation initiated a series of effects including local hyperthermia, immune response, production of oxygen reactive species, and elevated drug release, as shown in Figure 3. All of these have positive effects on killing cancer cells [9]. Gold nanoparticles [64], indocyanine green [65], reduced graphene oxide [66], iron-oxide [67,68], MoS_2_ [35], CuS [69], and black phosphorus quantum dots [36] have been reported as photo-responsive agents.

Using injectable hydrogel as depot, chemotherapy can be combined with photothermal therapy (PTT). An injectable in situ forming hydrogel was prepared based on chitosan to integrate IR780 as PTT agent and DOX as drug agent, both of which were loaded on polymeric nanoparticles to be further embedded in the chitosan network. The obtained hydrogel exhibits increased temperature by around 9.1 °C and improved DOX release by 1.7 times upon the NIR exposure. It was enabling the reduction in cancer cell viability to 9% [33]. Chitosan-agarose hydrogels were synthesized in another work where graphene oxide (GO) and reduced graphene oxide (rGO) were selected as NIR-sensitive agents. Doxorubicin and Ibuprofen carried out chemotherapy against MCF-7 breast cancer cells. Importantly, when the hydrogels contained both photothermal therapy and chemotherapy agents, the cancer cell viability was decreased to 34%, which was much lower than the value without NIR. These results verify the synergistic outcome of the two therapies grouped together [34].

Another example was to utilize synthetic poly(N-acryloyl glycinamide-*co*-acrylamide) (PNAm) hydrogel to hold poly(dopamine)-loaded gold nanoparticles and DOX drug. This system was designed to suppress breast cancer recurrence [28]. In vivo photothermal performance induced by the nanoparticles indicates that such a hydrogel system was able to increase the temperature to 50.6 °C, whereas the single PNAm hydrogel with no photo-responsive agent only created a temperature rise of 2.3 °C. DOX release from the hydrogel upon NIR irradiation showed two-times increase in contrast to the hydrogel without NIR lighting [28].

Photothermal therapy is also combined with immunotherapy, as shown by an injectable hydrogel based on PDLLA-PEG-PDLLA co-polymers. This system was specialized in the application of the following three nanoparticles: indocyanine green, resiquimod (R848) and CPG ODNs. The first one acted as NIR responder while the latter two functioned as the activators of dendritic cells which played important roles in presenting the tumor antigens to the T cells in lymph nodes [18]. In response to the NIR, indocyanine green increased the temperature to 45 °C to initiate thermal therapy and also promote the release of these two immune active particles at gel–sol transition. The proposed combination aimed to prohibit the recurrence and metastases of breast tumors, and using the injectable hydrogels not only achieved regionalized release, but also improved the aqueous stability and degradation problems of the three molecules [18]. Immunotherapy recently attracts much interest, and the primary goal is to activate the tumor-specific T cells. The use of scaffold implant to deliver the tumor-responsive T cells has been reported. Compared to intravenous and local injection of the T-cells, the implant system was able to support the proliferation of the T-cells that respond to the tumor in the resection zone and also suppress the cancer relapse. This discovery provides another evidence showcasing the positive effect of localized delivery of anticancer agents on cancer eradiation [70,71].

Furthermore, the combination of three cancer therapies has also been made possible to achieve chemotherapy, photothermal therapy, and photodynamic therapy in one system. Two-dimensional nanosheets, MoS_2_, were used as the DOX carrier and then entrapped in a polypeptide hydrogel based on PC_10_A to target 4T1 breast cancer cell. MoS_2_ exhibited dual roles in photothermal and photodynamic effect. It responded to NIR irradiation to enhance local hyperthermia and DOX release. It also produced oxygen reactive species for photodynamic therapy. This hybrid hydrogel containing multi-therapies was reported to generate improved in vivo antitumor effect compared to the single therapy [35]. Another hydrogel system was prepared by mixing negatively charged xDNA and positively charge polyethyleneimine particles with its surface functionalized by black phosphorus quantum dots (BPQD). BPQD was to facilitate both NIR triggered photothermal and photodynamic therapy while the DOX was loaded onto polyethyleneimine particles. It was found that this system was capable of improving the drug penetration to the tumors, survival rate of breast cancer-bearing mice, and drug resistance [36].

### 2.4. Thermo-Magnetic Dual Responsiveness

Thermo-magnetic dual responsive hydrogel is shown to increase the chemotherapy efficacy by the heat generation induced by magnetocaloric effect. Fe3O_4_ nanoparticles were introduced to the hydrogel network and subject to alternating magnetic field to yield heat and local hyperthermia. In response to the temperature increase, the hydrogels underwent reversible gel–sol transition when it was heated [40]. As reported, the injectable dual responsive hydrogel based on PEGylated Fe_3_O_4_ nanoparticles and α-cyclodextrin could host the following two anticancer drugs: hydrophobic paclitaxel (PTX) and hydrophilic doxorubicin (DOX). The hydrogel turned to liquid upon magnetically triggered hyperthermia and filled the post-surgery wound to disable the recurrence of breast cancer [40].

In a study regarding triple negative breast cancer, glycol chitosan and Fe3O_4_ nanoparticles were used to prepare the hydrogels, which could realize the dual release of the following two anticancer agents: doxorubicin and docetaxel. The use of this dual responsive hydrogel system showed the enhancement in antitumor efficacy and the reduction in cancer cell viability [19]. Another research group [41] reported a hydrogel system consisting of chitosan and β-glycerophosphate. This hydrogel incorporated super-paramagnetic graphene oxide, which was modified by polyethyleneimine and also carrying DOX. The system demonstrated improved MCF-7 cancer cell inhibition under the alternating magnetic field which induced the hyperthermia.

### 2.5. Glutathione and pH-Responsive Hydrogels

Drug deliveries responsive to glutathione concentration have been increasingly reported [72]. Combining these two pathophysiological characteristics as stimuli, pH-glutathione sensitive hydrogels were proposed. In such systems, in addition to pH-sensitive moieties, these hydrogels possess glutathione-reactive groups (e.g., disulfide) which accept the electrons from the thiol groups in glutathione. Thus, the hydrogel networks containing glutathione-reducible groups will degrade at tumor site to facilitate drug release [37,72]. Peptide-based hydrogels with pre-designed amino acid sequence were prepared via a self-assembly technique to incorporate anticancer drug paclitaxel (PTX). As shown by drug release test, PTX release rate increased in acidic solution and increased in the presence of glutathione [37]. Aza-Michael addition reaction was carried out to link PEGDA and polyamidoamine dendrimers to form the hydrogels which possessed pH-sensitive acetal group and glutathione-reducible disulfide linkages. The obtained hydrogels show increased swelling ratio and burst release of DOX in the pH of 3 and 5 than in physiological 7.4, and in the presence of DTT (glutathione substitute). The hydrogel degradation was enhanced in lower pH and higher concentration of DTT [39].

### 2.6. pH-Temperature Dual Responsiveness

The injectable hydrogel systems responding to both pH and temperature stimulation were reported more than a decade ago to load paclitaxel against breast cancer. In the work, the hydrogel was based on poly(ε-caprolactone-*co*-lactide)-poly(ethylene glycol)-poly(ε-caprolactone-*co*-lactide) block copolymer coupled with sulfamethazine oligomer [73].

More recently, isopropylacrylamide (NIPAAm), was reported to co-polymerize with pH-responsive itaconic acid to form poly(NIPAAm-*co*-IA). The resultant copolymer showed double responsiveness and could be further blended with chitosan through ionic crosslinking. The final hydrogels exhibited injectability, pH-temperature sensitive swelling ratios, and much-increased DOX release at acidic medium (pH = 5.5) compared to physiological 7.4 to eradicate breast cancer MCF-7 cell lines [42].

**Figure 4:**
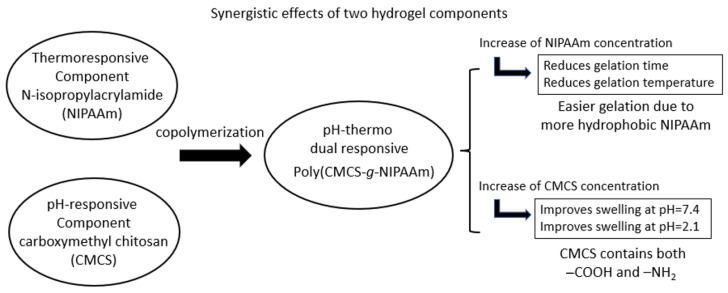
Synergistic effect of a pH-thermo dual responsive hydrogels composed of copolymer of poly(CMCS-*g*-NIPAAm).

NIPAAm can also be grafted onto carboxymethyl chitosan (CMCS) to form poly(CMCS-*g*-NIPAAm). Both NIPAAm and CMCS contents in the overall hydrogel system played a role in adjusting the gelation temperature and gelation time. Increasing the NIPAAm concentration decreased both sol–gel transition temperature and gelation time whereas the increase in CMCS content rose the gelation temperature and prolonged gelling time [43]. It was also important to note that CMCS resulted in the increase in swelling ratio of the hydrogel in both pH = 2.1 and pH = 7.4. This was attributed to CMCS being amphoteric containing both –COOH and –NH_2_ groups. 5-Fluorouracil was loaded to the hydrogel for breast cancer line; MCF-7, and its release rate was elevated by reducing the temperature from 37 °C to 25 °C and decreasing the pH from 7.4 to 2.1 [43]. Figure 4 has been drawn by the authors of the present review to illustrate the synergistic effect of this co-polymer. Instead of performing co-polymerization, chitosan, sodium-glycerophosphate and hyaluronic acid can be directly mixed to form injectable and dual responsive hydrogels. The addition of hyaluronic acid was reported to not only improve the mechanical properties of the chitosan (hydrogen bond formation) but also to reduce the burst release. Hydrogen bonding was formed between the carboxyl group of hyaluronic acid and protonated amines of chitosan to give a mechanically stronger network which in turn reduced the burst drug release of chitosan alone hydrogel (over 90% of DOX released during 20 h) [10]. All these examples discussed here indicate the synergistic effect between the components of the overall hydrogel system.

## 3. Biocompatibility

The injectable hydrogels reviewed in the present work aim to provide a drug release platform for breast cancer treatment. Thus, the most critical issue is to investigate the biocompatibility of the prepared hydrogels, especially for some nanoparticles and other functional agents [3] incorporated in the hydrogel networks.

Quantum dots produced from cadmium selenide have been found to be cytotoxic [74]. One research group has developed graphene-based iron oxide quantum dots and used them in the injectable drug delivery with good biocompatibility [67]. Black phosphorus quantum dots and polyethyleneimine have been applied to prepare the injectable hydrogels with xDNA. Polyethyleneimine is known to be non-biocompatible, but the modified hydrogel system exhibited low hemolysis rate, suggesting an improved biocompatibility [36]. These two examples indicate it is still feasible to utilize some non-biocompatible materials for drug delivery fabrication after necessary modification and careful cytocompatibility measurements.

However, sufficient attention and necessary biocompatibility tests are required for the hydrogel systems where polymerizations take place, as the system may contain unreacted toxic crosslinkers [75,76] and monomers [77], even though those agents are usually used with small amount.

## 4. Tough Hydrogels with Excellent Load-Bearing Performance

Injectable hydrogels for anticancer drug delivery need to remain in human body for a sustained period of time. In this regards, mechanical toughness and stability are important as the hydrogels must be able to sustain the external stress caused by body movement. Particularly for females, the support for breast movement by surrounding skins, tissues, and ligaments is limited [78]. Exercise and physical activities usually lead to breast displacement and pains [79,80,81]. Therefore, it is crucial for the injectable breast implants to be mechanically tough and stable. Owing to their large water content, conventional hydrogels are mechanically weak [82], which restricts their realization for biomedical applications including the load-bearing drug deliveries. However, many research works reviewed in the present work show limited focus on the toughness tests of the hydrogel samples. This is the remaining and challenging issue that require more research efforts and multidisciplinary collaborations. It is necessary to give a brief outline on the recent advancement in the tough hydrogels that may fill this gap.

**Figure 5:**
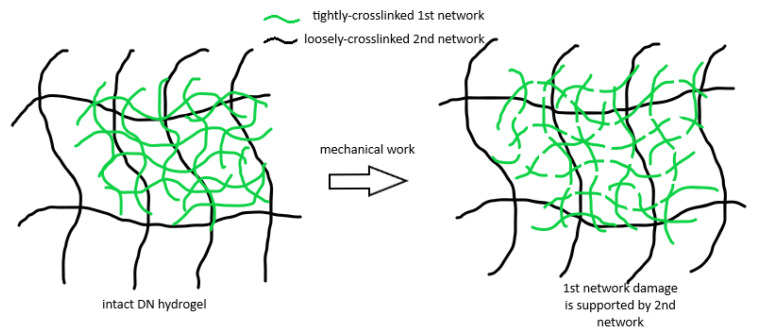
In DN hydrogel, the damage in the tightly crosslinked 1st network is supported by the loosely-crosslinked 2nd network, thus exhibiting no catastrophic failure of the overall DN hydrogel system.

During the past decade, a number of mechanically tough hydrogels have been reported, including double network (DN) hydrogels [82], ionic-covalent hybrid hydrogels [83], polymer-clay nanocomposite hydrogels [84], slide-ring hydrogel [85], and hydrogen-bonded polyurethane hydrogels [86,87]. These tough hydrogel systems not only possess high water content and biocompatibility but exhibit high mechanical strength (a few MPa) and toughness (10^2^~10^4^J/m^2^) as well. These results imply that these tough hydrogels are as tough as natural rubbers [83,88,89,90]. Many synthetic and natural polymers are used in the preparation of these tough hydrogels such as poly(vinylpyrrolidone) [91], poly(acrylamide) [92], poly(acrylic acid) [93], poly(*N*-isopropylacrylamide) [94,95], poly(2-acrylamido-2-methylpropanesulfonic acid) [82], poly(ethylene glycol) [96], alginate [83], agarose [97], and chitosan [98]. The first generation of double network (DN) hydrogels as reported by Gong and co-workers utilized covalent crosslinkers. The large fracture energy originates from the energy dissipated by the breakage of tightly crosslinked first network and those damages are supported by the loosely-crosslinked second network [99]. The essential shortcoming of this system is irreversible mechanical damage when the hydrogel is subject to external forces. This is evidenced by successive softening and irrecoverable hysteresis in series stretch/retract cycles [100]. As shown in Figure 5, in a DN hydrogel system, the first polymeric network is highly crosslinked and subject to fracture under external stress. Those fractured bonds absorb the mechanical energy, thus increasing the fracture toughness of the entire DN system. However, the damage created in the first network is supported by the loosely-crosslinked second polymeric network, which prevent the overall DN hydrogel from catastrophic failure [99,100]. From point of view of the authors for the present review, this design strategy may be useful for injectable hydrogels for breast cancer implants. The polymers that are sensitive to the glutathione, pH, photo, and other stimulus in the tumor site may be used as the first network which degrade and release the drug in response to the stimulations. Meanwhile, the damaged first network will still be held by the loosely-crosslinked second network to guarantee the structural integrity and to inhibit the burst release.

The essential weakness of the covalently crosslinked DN hydrogel is modified by ionic-covalent hybrid hydrogels which contains physically crosslinks. Such physically crosslinked tough hydrogels exhibit recoverability of mechanical damage, which overcomes the shortcomings of covalently crosslinked DN hydrogels since the fractured covalent bonds are not recoverable [101,102]. The recoverability of those physically crosslinked hydrogels significantly expands their potential as load-bearing drug delivery devices which need to sustain the human body movement for a set period of time.

Amongst physically crosslinked tough hydrogels, ionic-covalent hybrid hydrogels offer some of the highest toughness and stretchability [83,101,103]. In addition, such hybrid tough hydrogels could be ionic sensitive. For instance, in polyacrylamide-alginate ionic-covalent hybrid system, alginate is physically crosslinked by Ca^2+^ to form a special “egg-box” structure and its mechanical properties are tunable with the concentration of Ca^2+^ thus this hydrogel system is ionic sensitive [104].

The polyethylene glycol-based polyurethane (PEG-PU) hydrogels are also reported for the fabrication of tough hydrogels which exhibit a fracture energy of approximately 3 kJ/m^2^ and their enhanced mechanical properties are attributed to the hydrogen bonding formed by the urethane segment of PEG-PU [86,87]. PEG-PU possesses hydrophobic hard segments and hydrophilic polyethylene glycol soft segments, and has been adopted for injectable hydrogel drug delivery to release functional ions and drugs for tissue healing [105].

Nevertheless, the drawback of the tough hydrogels which are physically crosslinked by ionic chelation and hydrogen bonding is their time-dependent stress relaxation when it is loaded [88,106]. This mechanical instability may lead to a disrupted drug delivery when the implanted hydrogel system is under the stress created by body movements. Necessary research is needed to address this problem. One possible solution to solve this problem may rely on the applications of the tough hydrogels with fast recovery properties which can avoid prolonged network damage caused by the dissociations of physical crosslinks. Slide ring hydrogels may be a potential candidate. The hydrogels are composed of linear PEG chains crosslinked by ring-shaped hydroxypropyl-α-cyclodextrin (α-CDs) which are able to slide along the polymeric chains. In cyclic extension-relax tests, recoverable energy dissipation was observed owing to the orientation and dis-orientation of the PEG chains mediated by those moveable ring crosslinkers [107]. In another work, injectable double network hydrogels were prepared utilizing the guest-host association. The obtained hydrogels exhibit injectability, mechanical strength, seal-healing, and fast damage recovery [108]

In summary, this section provides a brief outline on the recent development in tough hydrogels which demonstrate superior mechanical toughness around a few kJ/m^2^. Physically crosslinked tough hydrogels are damage-recoverable but mechanically instable, which may limit their applications for sustained drug delivery under the physical body movement. Researchers have prepared the hydrogels crosslinked with sliding rings or gust-host complex to achieve the rapid damage recovery. This progress may offer possible answers to realize the tough hydrogels as load-bearing drug deliveries.

## 5. Summary and Prospect

The present review critically analyzes the application of stimuli-responsive hydrogels used as injectable drug delivery for breast cancer therapy. Among various response categories, temperature-triggered sol–gel–sol transition, pH-initiated drug release, photo-induced combinational therapies, magnetic-temperature dual responsiveness, pH-glutathione sensitivity and pH-temperature sensitivity are discussed. Meanwhile, the progress in tough hydrogel research is also briefly discussed as it is an encouraging achievement to overcome the mechanical weakness of many conventional hydrogel systems proposed for drug delivery. Based on the discussion in this review, the drug delivery devices using injectable hydrogel present many improvements over the conventional systemic chemotherapy such as controllable and sustained release, localized drug delivery, and enhanced therapy efficacy. All of this progress contributes to the reduction in side effects caused by prolonged chemotherapy administrated with conventional approaches.

The current trend and focus in this area are three-fold, as follows: (1) selection and combination of two or more smart polymers to fabricate dual and multiple-responsive hydrogels; (2) incorporation of a diverse range of nanoparticles or other molecules to produce a hydrogel with hybrid structures; (3) integration of multiple cancer treatment approaches into one delivery system to fulfill chemotherapy, immunotherapy, photothermal therapy, and photodynamic therapy simultaneously for elevated treatment efficacy. All these three aims can only be accomplished by ever-evolving design, preparation, and characterization of novel injectable hydrogels which function as both drug depot and smart platform in response to the change of the microenvironment of breast cancer site. Multidisciplinary collaborations are required to link conceptualization, material development, biological evaluation, prototyping, and clinical trials. Various skill and knowledge sets are also needed to engaged researchers with a background in materials characterizations, pharmacology, pharmacokinetics, and physiology.

One of the remaining challenges is the mechanical weakness of many hydrogels, which may limit and delay the realization of the hydrogels for many biomedical applications. The emergence of tough hydrogels supplies a hopeful answer for this issue, since those hydrogels demonstrate mechanical toughness, damage recoverability, and biocompatibility. However, the drawback of covalently crosslinked hydrogel is that it is not damage recoverable, while the physically crosslinked hydrogels exhibit stress relaxation and mechanical-instability. These may disrupt the drug release under the stress created by muscle and bone motions. Promising progress to overcome this challenge has been made. Further understanding on the structure–properties relationship of these novel and strong hydrogels is needed, and experimental investigations of utilizing the tough hydrogels in proposed biomedical applications should be carried out.

## Figures and Tables

**Table 1:** Stimuli-responsive injectable hydrogel drug delivery systems reported for breast cancer therapies. Multiple therapies can be embraced into one hydrogel system, including photothermal therapy (PTT), photodynamic therapy (PDT), immunotherapy, and chemotherapy.

Stimuli	Hydrogel Polymers	Nanoparticles	Anticancer Agents	Therapies	Cell Lines	Ref.
Temperature	(PLGA-PEG-PLGA) triblock copolymer		Herceptin antibody	Antibody therapy	SK-BR-3, breast cancer cell line	[21]
Temperature	Hyaluronic acid-chitosan-***g***-polyNIPAm	Folate-conjugated graphene oxide	Doxorubicin	chemotherapy	MCF-7, human breast cancer	[25]
Temperature	Gellan	Prussian blue nanoparticles	Prussian blue nanoparticles and combretastatin A4	Combined PTT and tumor vascular disruption	4T1 mouse breast cancer	[26]
Temperature	Pluronic	TMC loaded niosomes	tamoxifen citrate (TMC)	chemotherapy	Ehrlich carcinoma cell line	[27]
Temperature-photo	poly(D,L-lactic acid)-poly(ethylene glycol)-poly(D,L-lactic acid) copolymers		indocyanine green resiquimod 484 CPG ODNs	Combined PTT and immunotherapy	4T1 mouse breast cancer cell line	[18]
Temperature-photo	poly(***N***-acryloylglycinamide-***co***-acylamide)	polydopamine coated-gold nanoparticles	Doxorubicin	PTT and chemotherapy	4T1 breast cancer	[28]
pH	chitosan-***g***-dihydrocaffeic acid	Oxidized pullulan	Doxorubicin	chemotherapy	HCT116 human Colon tumor cell	[29]
pH	glycol chitosan (GC)-Pluronic F127		Doxorubicin	chemotherapy	H22 hepatocellular carcinoma cell line [30]	[31]
pH	Based on gelatin and PEG	Laponite nanodisks	Doxorubicin	chemotherapy	MCF-7 human breast cancer cell line and MDA-MB-231 triple negative breast cancer cell line	[23]
pH	Silk nanofiber hydrogel		Doxorubicin	chemotherapy	MDA-MB-231 human breast cancer	[32]
Photo-	Low melting point agarose	MnO_2_	Sodium humate and chlorin e6	Combined PTT and PDT	4T1, murine breast cancer	[22]
Photo-	Crosslinked chitosan	IR780 loaded polymer nanoparticles and DOX-loaded PEG nanoparticles	Doxorubicin	Combined PTT and chemotherapy	MCF-7 breast cancer cell line	[33]
Photo-	Chitosan and agarose	Graphene oxide and reduced graphene oxide	Doxorubicin and ibuprofen	Combined PTT and chemotherapy	MCF-7 breast cancer cell line	[34]
Photo-	Peptide based hydrogels	2D MoS_2_ nanosheet	Doxorubicin	Combined PTT, PDT, chemotherapy, and immunotherapy	4T1 breast cancer	[35]
Photo-	DNA based hydrogels	polyethyleneimine particles functionalized by black phosphorus quantum dots	Doxorubicin	Combined PTT, PDT and chemotherapy	MCF-7 breast cancer	[36]
pH and glutathione	Peptide based hydrogel with pH and glutathione sensitivity		paclitaxel	chemotherapy	MCF-7 human breast cancer and 4T1 murine breast cancer	[37]
pH and glutathione	PEGDA and polyamidoamine dendrimers		Doxorubicin	chemotherapy	HeLa cells Cervical cancer cell line [38]	[39]
Thermo-Magnetic	Self-assembled PEGylated Fe_3_O_4_ nanoparticles and α-CD	PEGylated Fe_3_O_4_ nanoparticles	Paclitaxel and doxorubicin	Combined hyperthermia and chemotherapy	4T1 breast cancer	[40]
Thermo-Magnetic	glycol chitosan crosslinked with DF-PEG-DF	Fe_3_O_4_ nanoparticles	Doxorubicin and docetaxel	Combined hyperthermia and chemotherapy	MDA-MB-231 breast cancer	[19]
Thermo-Magnetic	chitosan and β-glycerophosphate	graphene oxide modified by polyethyleneimine	Doxorubicin	Combined hyperthermia and chemotherapy	MCF-7	[41]
pH-temperature	poly(***N***-isopropylacrylamide-***co***-itaconic acid) blended with chitosan		Doxorubicin	chemotherapy	MCF-7 human breast cancer	[42]
pH-temperature	poly(CMCS-***g***-NIPAAm)		5-Fluorouracil	chemotherapy	MCF-7 breast cancer	[43]
pH-temperature	chitosan, sodium-glycerophosphate and hyaluronic acid		Doxorubicin	chemotherapy	Hela cells Human cervical cancer cell	[10]

## Data Availability

Not applicable.
